# Coexistence of the Band Filling Effect and Trap-State Filling in the Size-Dependent Photoluminescence Blue Shift of MAPbBr_3_ Nanoparticles

**DOI:** 10.3390/nano14191546

**Published:** 2024-09-25

**Authors:** Jing Sun, Mengzhen Chen, Tao Huang, Guqiao Ding, Zhongyang Wang

**Affiliations:** 1 Shanghai Advanced Research Institute, Chinese Academy of Sciences, Shanghai 201210, China; sunj@sari.ac.cn (J.S.); mengzhen_chen@gtasemi.com.cn (M.C.); 2 University of Chinese Academy of Sciences, Beijing 100049, China; 3 Department of Material Science and Engineering, Southern University of Science and Technology, Shenzhen 518000, China; huangt@sustech.edu.cn; 4 National Key Laboratory of Materials for Integrated Circuits, Shanghai Institute of Microsystem and Information Technology, Chinese Academy of Sciences, Shanghai 200050, China

**Keywords:** MAPbBr_3_ perovskite nanoparticles, photoluminescence blue shift, quantum confinement effect, band filling effect, trap-state filling

## Abstract

The size-dependent photoluminescence (PL) blue shift in organometal halide perovskite nanoparticles has traditionally been attributed to quantum confinement effects (QCEs), irrespective of nanoparticle size. However, this interpretation lacks rigor for nanoparticles with diameters exceeding the exciton Bohr radius ($r_{B}$). To address this, we investigated the PL of MAPbBr_3_ nanoparticles (MNPs) with diameters ranging from ~2 to 20 nm. By applying the Brus equation and Burstein–Moss theory to fit the PL and absorption blue shifts, we found that for MNPs larger than $r_{B}$, the blue shift is not predominantly governed by QCEs but aligns closely with the band filling effect. This was further corroborated by a pronounced excitation-density-dependent PL blue shift (Burstein−Moss shift) at high photoexcitation densities. Additionally, trap-state filling was also found to be not a negligible origin of the PL blue shift, especially for the smaller MNPs. The time-resolved PL spectra (TRPL) and excitation-density-dependent TRPL are collected to support the coexistence of both filling effects by the high initial carrier density (~10^17^–10^18^ cm^−3^) and the recombination dynamics of localized excitons and free carriers in the excited state. These findings underscore the combined role of the band filling and trap-state filling effects in the size-dependent PL blue shift for solution-prepared MNPs with diameters larger than $r_{B}$, offering new insights into the intrinsic PL blue shift in organometal halide perovskite nanoparticles.

## 1. Introduction

Halide perovskite nanoparticles have garnered significant attention over the past decade due to their high photoluminescence quantum yields, exceptional charge mobility, and superior color purity, which make them ideal candidates for optoelectronic devices such as solar cells [1,2], light-emitting diodes [3], and photodetectors [4]. Compared to traditional semiconductors, halide perovskite nanoparticles offer several key advantages, including tunable emission properties, low production costs, and straightforward processing techniques, all of which position them as promising materials for various applications. To achieve tunable emission, extensive efforts have been made to control the bandgap shift by manipulating factors such as particle size, surface ligand ratio [5], composition [6], and by adjusting the temperature or excitation intensity [7]. Among these, size control has been frequently employed to induce a photoluminescence (PL) blue shift, typically attributed to quantum confinement effects (QCEs) [6,8]. For instance, Quinten et al. reported that CsPbBr_3_ nanoplatelets, with thicknesses tunable from 1.83 nm to 2.98 nm, exhibited strong two-dimensional carrier confinement, resulting in a bandgap blue shift exceeding 0.47 eV compared to bulk CsPbBr_3_ [9]. Similarly, Butkus et al. observed that the emission peak of halide perovskite nanocrystals shifted from 516 nm to 495 nm as their size decreased from ~9 nm to ~4 nm, attributing the shift to the QCE [10]. Generally, the QCE is effective in adjusting the bandgap when nanoparticle sizes are smaller than the exciton Bohr radius ($r_{B}$), which for perovskites has been reported to range between 2 and 4 nm [5,8]. However, for perovskite nanoparticles with diameters larger than $r_{B}$, attributing the observed PL blue shift solely to the QCE lacks rigor [11,12], necessitating a more explicit understanding of the underlying mechanisms.

Apart from the QCE, the band filling effect (Burstein–Moss effect) is often invoked to explain the pronounced PL blue shift observed in traditional semiconductor nanoparticles under conditions of high doping or strong excitation [7,13,14]. In such scenarios, the nanoparticles exhibit high carrier densities, resulting in band filling close to the conduction band [15]. For example, Yang et al. reported a 90 meV blue shift in the luminescence of pencil-like ZnO nanowires, which was attributed to the band filling effect under a high carrier density, and they proposed a relationship between the blue shift and size in the range of 700 to 50 nm [16]. In the case of organometal halide perovskites, similar studies have been conducted on two-dimensional materials at the hundred-nanometer scale. Wang et al. observed a blue shift in the transient PL spectra of MAPbI_3_ films as the excitation density increased from 1.9 × 10^16^ cm^−3^ to 2.9 × 10^18^ cm^−3^, attributing this shift to the band filling effect [17]. Similarly, Manser et al. investigated the excited-state dynamics at the band edge of MAPbI_3_ films, demonstrating that the band edge shifts as a function of charge carrier density above a certain onset threshold of 7.5 × 10^17^ cm^−3^ [7]. Interestingly, this threshold correlates with the trap density, which can serve as a measure of the material’s trap density. Surface defect states, often introduced by surface ligands and environmental conditions during synthesis [18], commonly accompany both the QCE and the band filling effect, playing a crucial role in blinking and exciton recombination dynamics [19,20,21]. Although previous research has established that a high carrier density can induce the band filling effect in crystals on the scale of hundreds of nanometers, these studies have largely overlooked nanoparticles with sizes slightly larger than $r_{B}$. This motivated our investigation into the PL blue shift of MAPbBr_3_ nanoparticles (MNPs) with sizes larger than $r_{B}$, specifically focusing on the band filling effect under a high excitation density.

In this study, we aimed to explore the origin of the PL blue shift in MNPs with sizes around and larger than $r_{B}$, while minimizing sample variability apart from size. To this end, we prepared MNPs with diameters (*R*) ranging from ~2 nm to ~20 nm from the same batch using ultra-high-speed centrifugation. Our findings reveal that for MNPs larger than $r_{B}$, the QCE is not the predominant mechanism driving the PL blue shift, as demonstrated by theoretical fitting based on the Brus equation. Instead, the PL emission energy and bandgap energy were found to be linearly proportional to $R^{-2/3}$, consistent with the Burstein–Moss theory, thereby indicating the dominance of the band filling effect. Additionally, through the excitation-density-dependent PL blue shift, trap-state filling was also found to be not a negligible origin of the PL blue shift, especially for the smaller MNPs. The time-resolved PL spectra (TRPL) and excitation-density-dependent TRPL are collected to support the coexistence of both filling effects by the high initial carrier density (~10^17^–10^18^ cm^−3^) and the recombination dynamics of localized excitons and free carriers in the excited state. These findings underscore the combined role of the band filling and trap-state filling effects in the size-dependent PL blue shift for solution-prepared organometal halide perovskite nanoparticles with sizes beyond $r_{B}$, with implications for their application, and offers new insights into the intrinsic origin of PL.

## 2. Materials and Methods

### 2.1. Synthesis of MAPbBr_3_ Nanoparticles (MNPs)

All materials and chemical reagents were purchased from Shanghai Titan Technology Corporation (Shanghai, China) and used as received without further purification. MAPbBr_3_ nanoparticles were synthesized using a one-step solution process, specifically the ligand-assisted re-precipitation strategy, as detailed in reference [22]. Initially, 0.16 mmol of MABr and 0.2 mmol of PbBr_2_ were dissolved in a mixture comprising 5 mL of N, N-Dimethylformamide (DMF), 0.05 mL of oleylamine, and 0.5 mL of oleic acid to form the precursor solution. Subsequently, 0.25 mL of the precursor solution was rapidly injected into 5 mL of toluene preheated to 60 °C under vigorous stirring. The reaction mixture immediately turned green-yellow, indicating the formation of MNPs, and the reaction was allowed to proceed for 5 min.

Size separation of the nanoparticles was achieved through prolonged high-speed centrifugation at 45,000 rpm for 6 h, 30,000 rpm for 6 h, and 10,000 rpm for 15 min, respectively. The samples were purified using a sequential centrifugation method: the first centrifugation at 45,000 rpm for 6 h resulted in three distinct layers. These layers were carefully separated and subjected to a second round of centrifugation. The top layer was designated as MNPs-S1. The middle layer was further centrifuged at 30,000 rpm for 4 h and separated into two parts, labeled MNPs-S2 and MNPs-S3. Finally, the bottom layer was centrifuged at 10,000 rpm for 15 min, yielding a supernatant designated as MNPs-S4 and a precipitate designated as MNPs-S5.

### 2.2. Characterization

All optical measurements were conducted at room temperature. UV–vis absorption spectra were acquired using a Cary 5000 UV–vis-NIR spectrophotometer with 1 cm quartz cuvettes. For PL measurements, the MNPs of various sizes were deposited onto SiO_2_/Si substrates. PL spectra were excited using a Ti: sapphire laser system (Chameleon Vision, Coherent Inc, PA, United States) with a repetition rate of 80 MHz and a wavelength of 375 nm. The spectra were collected using an iHR 550 Jobin Yvon spectrometer (HORIBA Scientific, Kyoto, Japan). The recombination dynamics were analyzed through time-correlated single-photon counting (HydraHarp 400, PicoQuant, Berlin, Germany), performed using the same Ti: sapphire system, and the PL lifetime measurements were carried out using a single-photon detector (PMA 185, PicoQuant, Berlin, Germany).

## 3. Results and Discussion

**Figure 1:**
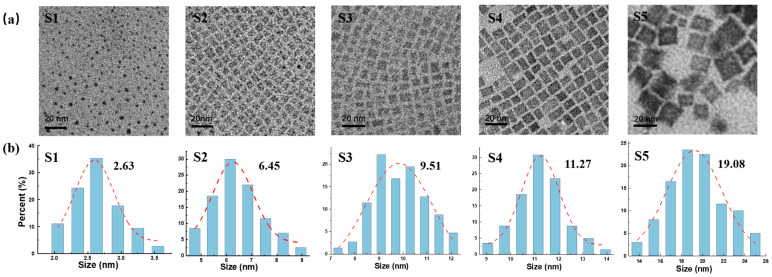
(**a**) TEM images of MNPs S1–S5. (**b**) The corresponding size distribution statistics histogram.

The MNPs were synthesized using a ligand-assisted re-precipitation strategy, as previously reported in reference [22]. To minimize sample variability, we obtained five nanoparticle samples of varying sizes from the same batch through ultra-high-speed centrifugation. High-resolution transmission electron microscopy (HRTEM) was employed to determine the size distribution and morphology of the MNPs. As illustrated in Figure 1, the five MNP samples, ranging in size from approximately 2 nm to 20 nm (denoted as S1–S5), exhibited a uniform size distribution (the as-prepared MNPs S1–S5 are shown in Figure S1 in the Supporting Information, SI). MNPs S1 displayed a spherical morphology, while MNPs S2–S5 exhibited cubic crystalline structures, consistent with previous reports [23,24]. The MNPs demonstrated high crystallinity, as evidenced by the lattice fringes observed throughout individual particles in the HRTEM images (see Figure S2a in SI). X-ray diffraction (XRD) analysis further confirmed the crystal structure of the MNPs, with the diffraction peaks sharpening as particle size increased (Figure S2b). The diffraction peaks for the variously sized MNPs aligned well with reports in the literature, confirming the crystallinity and purity of the synthesized MNPs [23].

**Figure 2:**
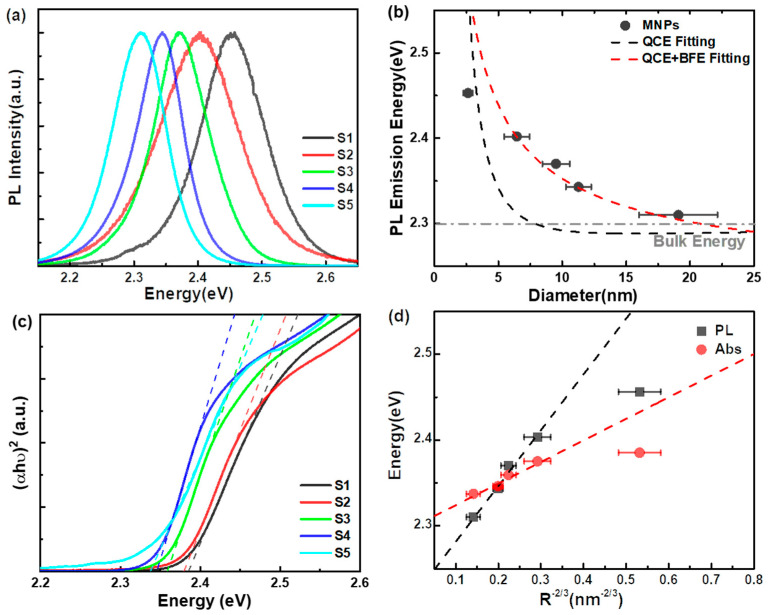
(**a**) PL spectra of MNPs S1–S5, excited by a 375 nm pulsed laser with a repetition rate of 80 MHz, excitation density is ~10 nJ/cm^2^. (**b**) PL emission energy as a function of the diameter of the MNPs: the black dashed curve is the theoretical fitting result based on Brus equation (QCE) and the red dashed curve is the integrated fitting result based on Brus equation and Burstein–Moss theory (QCE and band filling effect (BFE)). (**c**) UV–vis absorption spectra of MNPs S1–S5 and they were obtained from the absorption data by Kubelka–Munk transformation. The dashed lines are fitting tangents from the Tauc plot. (**d**) The bandgap energy obtained by absorption as a function of $R^{-2/3}$ of MNPs (red dashed line); the PL emission energy as a function of $R^{-2/3}$ of MNPs (black dashed line).

Due to the unintentionally doped defects (such as surface or internal defects) in MNPs S1–S5 in the solution preparing prosses, the MNPs were deposited onto a SiO_2_/Si substrate to further passivate the surface for testing the PL. The PL spectra of MNPs S1–S5, shown in Figure 2a, reveal a pronounced PL blue shift from 2.313 eV to 2.455 eV as the nanoparticle size decreases from 19.08 nm (S5) to 2.63 nm (S1). This size-dependent PL tunability has also been observed in other classical nano semiconductor materials [25,26]. The blue shifts are commonly attributed to the QCE, particularly when the particle size approaches the $r_{B}$. One of the most widely used models for describing the QCE is the Brus equation, which is based on the effective mass approximation [9,10,27], and is expressed as follows:

(1) $$E_{PL}=E_{g,bulk}+\frac{\hbar^{2}\pi^{2}}{2R^{2}}\frac{1}{m^{*}}-\frac{1.8e^{2}}{4\pi \varepsilon R}$$

Here, $E_{PL}$ is the PL emission energy of the nanoparticles, $E_{g,bulk}$ ≈ 2.3 eV is the widely accepted intrinsic bandgap of bulk MAPbBr_3_ [28], ℏ is the reduced Planck constant, R is the nanoparticle diameter, and $m^{*}$ is the reduced mass of the electron and hole, previously determined to be 0.13 [29,30]. The dielectric constant ε is calculated to be approximately 6.91 (detailed calculations are provided in the SI), consistent with literature values ranging from 4 to 10 (as detailed in SI Table S2) [28,29,30,31]. In this study, the values $E_{g,bulk}$ ≈ 2.3 eV and ε ≈ 6.91 were used and the fitting curve (black dashed curve) is displayed in Figure 2b. The fitting curve based on Equation (1) shows a sharp decrease around MNPs S1, with a size of 2.63 nm, and the theoretical PL emission energy approaches the bulk energy (~2.3 eV) as the nanoparticle size increases. However, the experimental data for MNPs S2–S5 deviate from the fitting curve.

According to the hydrogen-like model, the $r_{B}$ can be expressed as follows [29]: $r_{B}=\frac{\varepsilon }{m^{*}}a_{B}$, where $a_{B}$ is the Bohr radius of the hydrogen atom. Here, $r_{B}$ was calculated to be approximately 2.81 nm (detailed calculations are provided in the SI), which aligns with previously reported values in the range of 2 to 4 nm [28,29,30,31]. Therefore, the PL emission energy of S1, with a size of 2.63 nm, corresponds to the strong confinement regime, indicating that the QCE is the primary factor influencing the PL blue shift. For MNPs S2–S5, with sizes larger than $r_{B}$, the experimental data deviated from the theoretical fitting, suggesting the presence of additional contributing factors in the weak confinement regime.

The absorption spectra of the MNPs were recorded using a Cary 5000 UV–vis-NIR spectrophotometer at room temperature, as illustrated in Figure 2c. It is evident that the optical band edge shifts to lower energy as the nanoparticle size increases. The bandgap energy was determined from the absorption spectra using the Kubelka–Munk transformation [$F\left(r\right)={\left(1-r\right)}^{2}/2r=\alpha /S$], where *r* is the reflectance, *α* is the absorption coefficient, and *S* is the scattering coefficient. The bandgap energy ($E_{Abs}$) was identified at the intersection of the tangent line of the MNPs’ absorption curves and the abscissa. The fitting tangents, shown as dotted lines in Figure 2c, depict a blue shift from approximately 2.337 eV to 2.385 eV as the nanoparticle size decreases (see SI Table S1 for details).

Recent experiments have reported phenomena such as unintentional p–type or n–type doping behavior [32], elemental defects stemming from Frenkel defects (including Pb, I, and MA vacancies) [33], and a high trap density in solution-processed organometal halide perovskites [34], all of which highlight the significant unintentional doping present in these materials. In such cases, when the carrier density becomes sufficiently high, the Fermi level shifts into the conduction band (CB). As a result, the states within the CB below the Fermi level become occupied, prohibiting electron transitions from states below the Fermi level and leading to a widening of the bandgap. Consequently, the band filling effect is primarily considered responsible for the observed absorption blue shift in the MNPs. The blue shift energy ($∆E_{BM}$) associated with the Burstein–Moss band filling effect can be expressed as follows [7]:

(2) ∆EBM=ℏ22m*(3π2n)23

Here, n represents the carrier concentration, $n=\frac{F}{\hbar \omega_{0}R}\;$[13], F is the excitation density, R is the nanoparticle diameter, and $E_{Abs}=E_{g,Bulk}+∆E_{BM(Abs)}$, $∆E_{BM(Abs)}$ being the blue shift energy of absorption. Exciton diffusion is not considered in this analysis because the laser pulse width (140 fs) is much shorter than the exciton decay constant. The experimental data for $E_{Abs}\propto R^{-2/3}$, shown as the red dashed line in Figure 2d, demonstrate a strong correlation between the observed results and the band filling effect for samples S2 to S5, with the exception of S1. Additionally, we applied Equation (2) and $E_{PL}=E_{g,Bulk}+∆E_{BM(PL)}$ to explain the PL blue shift of the MNPs, ($∆E_{BM(PL)}$ represents the blue shift energy of PL. The relationship $E_{PL}\propto R^{-2/3}$ also showed a good linear fit in Figure 2d (black dashed line), except for S1.

In addition, considering the unintentional doping present in the MNPs during the solution process is near-equal, we thought the observed blue shift in absorption and PL may originate from QCE and the band filling effect at first, and not the defect state. To further validate this concept, we employed an integrated approach using Equations (1) and (2) to fit the PL emission energy as a function of the MNPs’ diameter (Figure 2b). Overall, the experimental data show a strong correlation with the fitted curve, particularly highlighting the band filling effect in MNPs S2–S5. 

**Figure 3:**
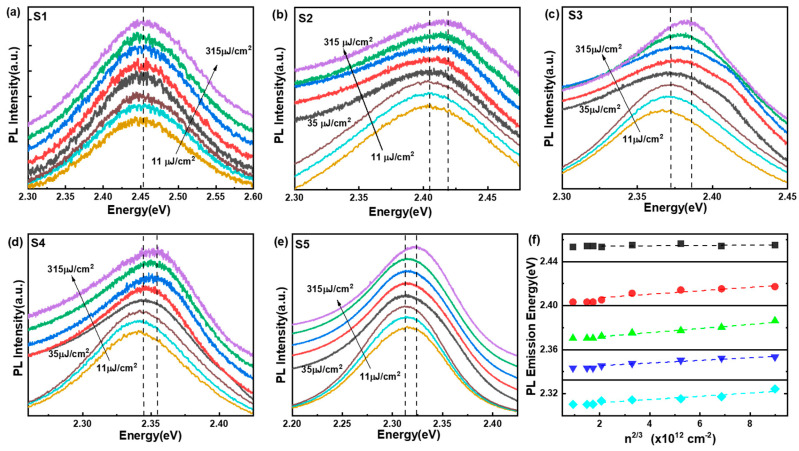
(**a**–**e**) Excitation-density-dependent PL of MNPs S1–S5. The excitation density increases from 11, 21, 27, 35, 70, 140, 210, to 315 μJ/cm^2^, respectively, and the excitation photocarrier concentration (n) is in the range of ~9.4 × 10^17^ cm^−3^ to 27 × 10^18^ cm^−3^. (**f**) PL emission energy of MNPs S1–S5 (top to bottom) versus $n^{2/3}$.

Due to the excitation-density-dependent PL blue shift associated with the band filling effect, we conducted further measurements of the PL spectra under varying excitation densities. The Mott criterion is defined as $n_{crit}=(\frac{k_{B}T}{E_{B}})/(11\pi r_{B}^{3})$ [35], where $k_{B}$ is the Boltzmann constant, T is the temperature, $E_{B}$ is the exciton binding energy, and $r_{B}$ is the exciton Bohr radius. The critical carrier concentration for MAPbBr_3_ is estimated to be in the range of 5.8 × 10^17^ cm⁻^3^ to 1.2 × 10^18^ cm⁻^3^, based on previously reported parameters ($E_{B}$ = 15.33 meV, $r_{B}$ = 4.38 nm [29]; $E_{B}$ = 76 meV, $r_{B}$ = 2 nm [30]; $E_{B}$ = 21 meV, $r_{B}$ = 3.74 nm [31]). Here, the critical carrier concentration of MNPs S1–S5 are 6.4 × 10^17^, 7.2 × 10^17^, 9.2 × 10^17^, 1.2 × 10^18^, and 1.4 × 10^18^ cm^−3^, respectively. Consequently, the excitation density was varied from ~11 μJ/cm^2^ to 315 μJ/cm^2^ to measure the PL spectra (corresponding excitation photocarrier concentrations (n): ~9.4 × 10^17^ to ~27 × 10^18^ cm^−3^, calculation details shown in the SI) [36], as shown in Figure 3a–e. When the excitation density was below 35 μJ/cm^2^, none of the PL spectra of MNPs S1–S5 exhibited a blue shift. However, as the excitation density increased beyond ~35 μJ/cm^2^, MNPs S2–S5 showed a clear PL blue shift. According to Equation (2) and $E_{PL}=E_{g,Bulk}+∆E_{BM(PL)}$, the linear fitting of PL emission energy as a function of $n^{2/3}$ is depicted in Figure 3f. The good fit of $n^{2/3}$-dependent PL blue shift directly verifies that the band filling effect captures the essential physics of blue shift in MNPs S2–S5. And linear fitting of MNPs S2–S5 show almost the same slope. This is probably because of band gap recombination (competing with the band filling effect or narrowing the band gap) [16,37], and ion-migration-induced new defects [38]. In contrast, MNPs S1 did not display an obvious PL shift with increasing excitation density, likely due to the dominant influence of a strong QCE. Additionally, PL peak energies of MNPs S1–S5 as functions of log (the excitation densities) is further given in SI Figure S3. The localized state (defect or trap states) filling was taken to explain the PL energy and well fitted nearly logarithmically with the excitation densities increasing. There was a strong linear correlation for MNPs S2 (R^2^ = 0.93), and an ordinary linear correlation for MNPs S3 (R^2^ = 0.87), S4 (R^2^ = 0.85), and S5 (R^2^ = 0.78) at all excitation densities. The unshifted PL of MNPs S1 agrees with the feature of weak localized excitons [36]. For the solution-processed MNPs, the localized trap states caused by disorder or defects will also be filled by increasing densities of photogenerated carriers. The excitation-density-dependent PL shift indicates the coexistence of band filling and trap-state filling.

**Figure 4:**
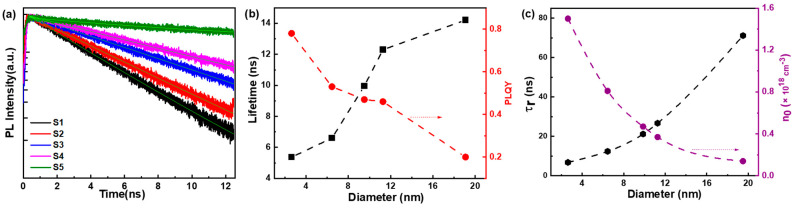
(**a**) The time-resolved PL spectra of MNPs S1–S5. (**b**) The trends in the average lifetimes and relative PLQY vs. the diameter. (**c**) The $\tau_{r}$ and initial carrier concentration ($n_{0}$) vs. the diameter.

Moreover, time-resolved PL spectra are commonly employed to calculate the carrier concentration. To further determine the initial carrier concentration in MNPs S1–S5, the time-resolved PL spectra were fitted using a single-exponential decay function, as shown in Figure 4a. The average lifetimes of MNPs S1–S5 are presented in Figure 4b, which indicate that the PL lifetime (τ) increases with particle size increasing, because surface trapping reduces as the surface/volume ratio decreases and the radiation lifetime increases with the increase in the radiation channel [39,40]. Subsequently, we calculated the relative photoluminescence quantum yield (PLQY) (Figure 4b, the detailed calculations are provided in the SI). According to the relationship between the PL lifetime and the relative PLQY, $\frac{1}{\tau }=\frac{1}{\tau_{r}}+\frac{1}{\tau_{nr}}$, $\tau_{r}=\frac{\tau }{PLQY}$, the non-radiative recombination lifetime ($\tau_{nr}$) and the radiative recombination lifetime ($\tau_{r}$) of MNPs S1–S5 were calculated, with the results shown in Table S3 and Figure 4c. The initial carrier concentration ($n_{0}$) was determined from the radiative recombination lifetime, $\tau_{r}\approx 1/(Bn_{0})$ [41], based on previous reports of the radiative recombination rate constant B (10^−10^ cm^3^/s) [42,43]. The initial carrier concentration of the MNPs was found to range from 1.4 × 10^17^ to 1.5 × 10^18^ cm^−3^ (results shown in SI Table S3). These values are consistent with the trap state density measurements in organometal halide perovskite nanocrystals (~10^17^–10^18^ cm^−3^) [34]. And the initial carrier concentration becomes higher as the diameter of the MNPs reduces, which means the smaller MNPs have more trap states and trap-state filling plays a more important role than band filling in smaller MNPs. This is consisted with the fact that MNPs S2 (R^2^ = 0.93) shows a better linear correlation than MNPs S3–S5 (R^2^ < 0.88) (SI Figure S3).

**Figure 5:**
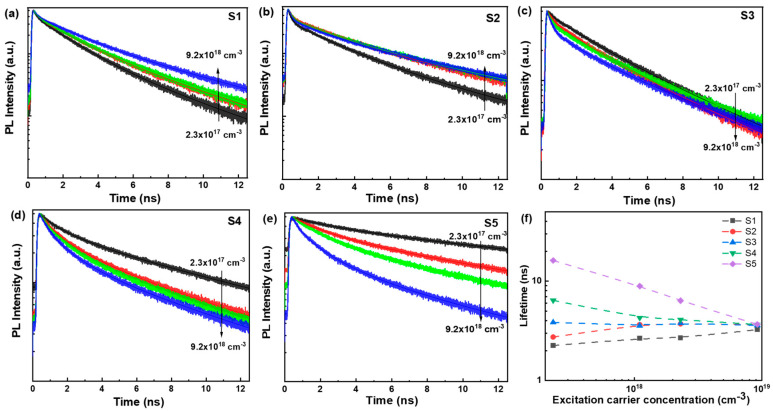
(**a**–**e**) Excitation-density-dependent time-resolved PL spectra. The excitation density increased from 2.7 μJ/cm^2^, 13.5 μJ/cm^2^, 27 μJ/cm^2^, to 81 μJ/cm^2^; the corresponding excitation carrier concentration: 2.3 × 10^17^ cm^−3^ (black line), 1.1 × 10^18^ cm^−3^ (red line), 2.3 × 10^18^ cm^−3^ (green line), 9.2 × 10^18^ cm^−3^ (blue line). (**f**) The average lifetime τ of MNPs S1–S5 vs. excitation carrier concentration.

To further validate the recombination dynamics of photogenerated carriers and gain deeper insight into the excited states, time-correlated single-photon counting was employed to analyze the recombination dynamics. The excitation density was varied from 2.7 μJ/cm^2^ to 81 μJ/cm^2^ (corresponding to excitation carrier concentrations of 2.3 × 10^17^ cm^−3^ to 9.2 × 10^18^ cm^−3^), covering the critical carrier concentration range. The excitation-density-dependent time-resolved PL spectra of MNPs S1–S5 are shown in Figure 5a–e. The PL decay curves of the MNPs were fitted using a double-exponential decay function expressed as $I\left(t\right)=A_{1}\exp\left(-t/\tau_{1}\right)+A_{2}exp\left(-t/\tau_{2}\right)$, where $A_{1}$ and $A_{2}$ are the amplitudes, and $\tau_{1}$ and $\tau_{2}$ are the corresponding lifetimes. The average lifetime (τ) is presented in Figure 5f.

For MNPs S1 and S2, as the excitation density increases, the decay dynamics slow down, and the lifetime slightly increases, suggesting that trap filling becomes the dominant process within this excitation density range due to the presence of larger defect states [44]. The slight increase in lifetime indicates that non-radiative Shockley–Read–Hall (SRH) recombination still dominates in small-sized perovskite nanoparticles under these excitation conditions and trap-state filling remains. A heavily doped InGaN/GaN quantum well shows a similar trend before the injected carrier density of 10^19^ cm^−3^ [45]. In contrast, for MNPs S3–S5, the decay dynamics accelerate as the excitation density increases, and the lifetime decreases with the rising excitation carrier concentration. This behavior can be interpreted as follows: as the injected carrier concentration increases, the defect states become filled and a large number of free electron–hole pairs accumulate at the band edge. It can be interpreted by second-order non-geminate/free carrier radiative recombination or three-body Auger recombination [45,46].

**Figure 6:**
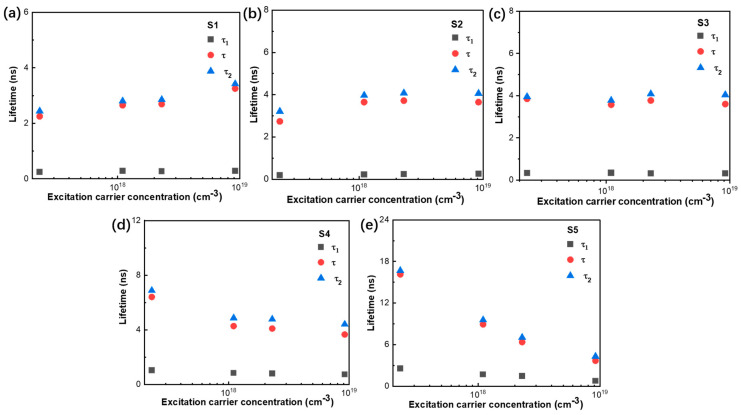
(**a**–**e**) The double-exponential-fitted PL lifetime $\tau_{1}$, $\tau_{2}$ and average lifetime (τ) vs. excitation carrier concentration (from 2.3 × 10^17^ cm^−3^, 1.1 × 10^18^ cm^−3^, 2.3 × 10^18^ cm^−3^ to 9.2 × 10^18^ cm^−3^) of MNPs S1–S5.

To gain deeper insight into the recombination processes, the trends in $\tau_{1}$, $\tau_{2}$, and the average lifetime (τ) of MNPs S1–S5 are presented in Figure 6. The short lifetime $\tau_{1}$ is almost independent of the excitation density for MNPs S1–S5. This suggests that $\tau_{1}$ is the trap-related nonradiative recombination (SRH recombination) [47], and almost independent of excitation density [48]. 

The radiative recombination lifetime $\tau_{2}$ and average lifetime (τ) of MNPs S4-S5 decreases with an increasing excitation density. This suggests exciton recombination and free carrier recombination in these MNPs, where the oscillator strength increases due to screening of the internal electric field under a high excitation density [49]. Meanwhile, the $\tau_{2}$ and τ of MNPs S3 are independent of excitation density, which is consistent with exciton recombination. And the slight increase in $\tau_{2}$ and τ for MNPs S1 and S2 indicates the process of trap filling [50]. The decrease in $\tau_{2}$ for S3–S5 arises from increased Coulombic screening and reduced binding energy due to a large number of photocarriers, leading to greater accumulation of free carriers at the band edge and the emergence of the band filling state. The faster decay led by Auger recombination is not observed here [46,51]. Auger recombination manifests under a high pump fluence (nonlinear regime), depending on the doping concentrations and excitation carrier density [45,46]. These excitation-density-dependent decay dynamics experiments provide strong evidence of trap-related nonradiative SRH recombination and free carrier recombination in the MNPs and indicate the coexistence of trap-state filling and the band filling effect in the excited states of the MNPs.

## 4. Conclusions

In conclusion, to explore the origin of PL blue shifts in MNPs near the $r_{B}$, we synthesized MNPs S1–S5 with sizes ranging from ~2 nm to 20 nm. As the nanoparticle size decreased, both absorption and the PL spectra exhibited a pronounced blue shift. Our analysis revealed that for MNPs with diameters larger than $r_{B}$, QCEs do not predominantly govern the PL blue shift. Instead, the band filling effect plays a significant role, as indicated by the linear relationship between PL emission energy and $R^{-2/3}$, and the observable blue shift with increasing excitation density from 35 μJ/cm^2^ to 315 μJ/cm^2^—except for MNPs S1, whose diameter is close to $r_{B}$. The calculated carrier density of ~10^18^ cm⁻^3^ induced by excitation was high enough to meet the theoretical criterion for the band filling effect (~10^17^–10^18^ cm⁻^3^). Additionally, the decay dynamics in the MNPs were examined using excitation-density-dependent time-resolved PL spectra. The short lifetime $\tau_{1}$, almost independent of excitation density for all MNPs S1–S5, indicates excitonic trapping recombination due to defect states. The trend in radiative recombination lifetimes $\tau_{2}$ with increasing excitation density in the MNPs further substantiates the presence of free carrier recombination and the coexistence of trap-state filling and the band filling effect in the excited state. These experiments underscore the critical contribution of trap filling and the band filling effect to the PL blue shift in solution-prepared MNPs with diameters ranging from 2 to 20 nm, providing new insights into the intrinsic origins of PL blue shifts and their potential applications.

## Data Availability

Data are contained within the article or the Supplementary Materials.

## Supplementary Materials

The following supporting information can be downloaded at: https://www.mdpi.com/article/10.3390/nano14191546/s1, Photographs of MNPs S1–S5 solution, the HRTEM image of crystal structure, XRD spectra, the PL emission energy, the bandgap energy and the binding energy, the calculation equation and methods of the exciton Bohr radius, the excitation photocarrier concentration, relative photoluminescence quantum yield, average PL lifetime, relative PLQY, radiative and non-radiative recombination lifetimes, initial carrier concentration and critical carrier concentration of MNPs are given in SI [52,53,54,55] cited in Supplementary Materials.
