# Supplementary material for: Coexistence of the Band Filling Effect and Trap-State Filling in the Size-Dependent Photoluminescence Blue Shift of MAPbBr3 Nanoparticles
Source: Nanomaterials (Basel). 2024 Sep 25;14(19):1546. doi: 10.3390/nano14191546 (PMC11477630; doi:10.3390/nano14191546)
Supplement: Supplementary file 1 [file nanomaterials-14-01546-s001.zip › nanomaterials-3187452-supplementary.pdf]

Supporting Information

Coexistence of the Band Filling Effect and Trap-State Filling in the Size-Dependent Photoluminescence Blue Shift of $MAPbBr_3$ Nanoparticles

Jing Sun [1,2,†], Mengzhen Chen [1,†], Tao Huang [3], Guqiao Ding [2,4,*] and Zhongyang Wang [1,2,*]

[1] Shanghai Advanced Research Institute, Chinese Academy of Sciences, Shanghai 201210, China; sunj@sari.ac.cn (J.S.); mengzhen_chen@gtasemi.com.cn (M.C.)
[2] University of Chinese Academy of Sciences, Beijing 100049, China
[3] Department of Material Science and Engineering, Southern University of Science and Technology, Shenzhen 518000, China; huangt@sustech.edu.cn
[4] National Key Laboratory of Materials for Integrated Circuits, Shanghai Institute of Microsystem and Information Technology, Chinese Academy of Sciences, Shanghai 200050, China
* Correspondence: gqding@mail.sim.ac.cn (G.D.); wangzy@sari.ac.cn (Z.W.)
† These authors contributed equally to this work.

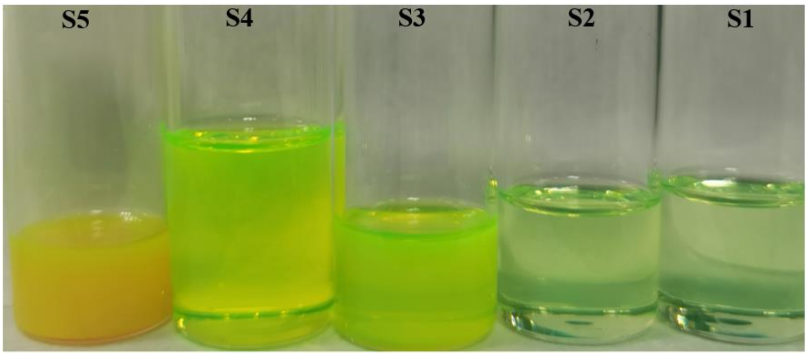

Figure S1. The photo image of MNPs S1-S5 separated from as prepared MNPs.

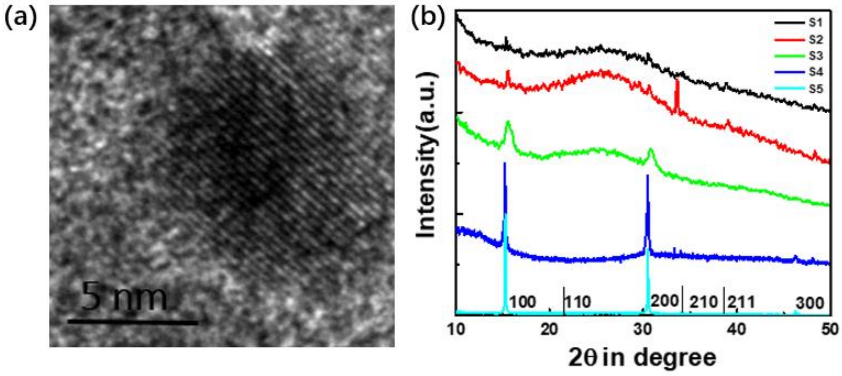

Figure S2. (a) The HRTEM image of a typical MNPs. (b) The XRD spectra of MNPs S1–S5.

Table S1. The PL emission energy (E_{PL}), the bandgap energy (E_{Abs}) obtained from the absorption spectra by Kubelka–Munk transformation, and the binding energy (E_B). The intrinsic bandgap of bulk $MAPbBr_3$ ($E_{g,bulk}$) set as 2.3 eV.

MNPs	PL emission energy (eV)	The bandgap energy (eV)	The binding energy (meV)
S1	2.455	2.385	85
S2	2.398	2.375	75
S3	2.370	2.359	59
S4	2.340	2.346	46
S5	2.313	2.337	37

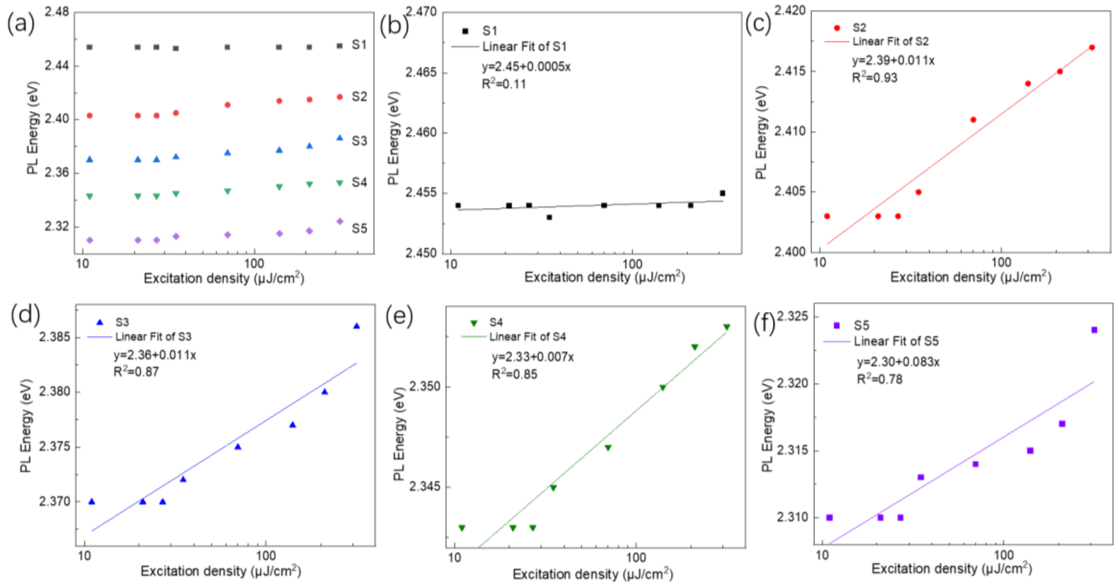

Figure S3. The PL peak energy versus log (excitation density) of all MNPs (a), S1 (b), S2 (c), S3(d), S4(e), S5(f).

The calculation of the exciton Bohr radius of MNPs

Considering low dimensionality dielectric confinement effect, the dielectric constant and the exciton Bohr radius can be emanated from the variables obtained so far, using the Hydrogen-like model relations [29]: $E_B = \frac{m^*}{\varepsilon^2} Ry(H), r_B = \frac{\varepsilon}{m^*} a_B$, $m^* = {}^{\mu}/_{m_0}$, here, μ is the exciton reduced mass ($0.13m_0$), m_0 is a free electronrest mass, $Ry(H) = 13.6eV$ is the hydrogen atom ground states energy and the a_B is the Bohr radius of the hydrogen atom (0.0529 nm), m^* is the reduced mass of the electron and hole of MNPs (0.13). The E_B of MNPs S5 as a reasonable fitting parameter to derived the value of ε = 6.91 and r_B =2.81 nm. Considering the previous reported data, see **Table S2**, the values are appropriate.

Table S2. Previous reported data of the parameters of the crystals $MAPbBr_3$.

Bohr radius/r_B (Å)	20 [(a)]	37.4±2.7[(b)]	4.38 [(g)]		
binding energy/E_B (meV)	76[(a)]	75[(c)]	80[(d)]	60[(f)]	320[(h)]
band gap/E_g (Ev)	2.28-2.29[(h)]	2.30[(b), (e)]			
dielectric constant/ε	4.8[(a)]	9.18[(b)]	7.5–9.8[(e)]	10.75[(g)]	

(a) Kenichiro et al. [30]; (b) T. Thu Ha Do et al. [31]; (c) Prashant K. et al. [52]; (d) Hailong H.et al. [53]; (e) Krzysztof G. et al. [28]; (f) Christoph et al. [54]; (g) Jenya Tilchin et al. [29]; (h) Kaibo Z. et al. [20].

The calculation of the excitation photocarrier concentration [36]:

Excitation photocarrier concentration (n) = light fluence density of a single pulse/(photo energy×optical penetration depth)=35 μJ/cm^2/(3.306 eV×1.6×10^{-19} J/eV ×220 nm) = 3×10^{18} cm^{-3}.

Here, the photon energy is 3.306 eV (excitation wavelength=375nm), the optical penetration depth of $MAPbBr_3$ is taken as ~220 nm [55].

Excitation density (μJ/cm^2)	11	21	27	35
	70	140	210	315
n (×10^{18} cm^{-3})	0.94	1.8	2.3	3
	6	12	18	27

The calculation of relative photoluminescence quantum yield

Reagent: Rhodamine 6G was selected as the comparative material and the solution was absolute ethanol, in which the PLQY of rhodamine 6G at an excitation wavelength of 488 nm reached 0.94 (Y_{R6}=0.94). The PLQY was not change with the excitation wavelength under the same solution. The solution of MNPs was toluene.

Instrument: The Cary 5000 absorption spectrometer was used for absorption spectroscopy. Self-built reflective PL spectra system that the light source is a Ti: sapphire system (Chameleon Vision, Coherent) with a repetition rate of 80MHz was used to measure the PL with 375nm laser.

Test: Excluding the influence of background noise and reagents on absorption, the absorption spectra of rhodamine 6G and MNPs were measured under the same condition, the test range is 600 nm to 300 nm. The solution was continuously diluted until the absorption intensity (A_{R6}, A_{MNPs}) at 375 nm is less than 0.05.

The PL spectra of rhodamine 6G and MNPs diluted were measured under the self-built reflective PL system under the same excitation wavelength and intensity. The influence of background noise and solution were excluded. The PL spectra range is 450nm to 600nm.

Calculation: The acquired PL spectra was integrated and divided into the integrated area (F_{R6}, F_{MNPs}). The relative PLQY was calculated by the following equation:

$$\mathrm{PLQY} = Y_{R6} \times \frac{F_{MNPs}}{F_{R6}} \times \frac{A_{R6}}{A_{MNPs}}$$

The radiative lifetime has been calculated according to the relationship between lifetime and relative PLQY: $\frac{1}{\tau} = \frac{1}{\tau_r} + \frac{1}{\tau_{nr}}$, $\tau_r = \frac{\tau}{PLQY}$, here, τ_r and τ_{nr} is radiative lifetime and non-radiative lifetime, respectively.

The calculation of initial carrier concentration (n_0) and the radiation complex factor B is 10^{-10} cm^3/s [42,43].

$$\tau_r = 1/(Bn_0)$$

Table S3. Average PL lifetime, relative PLQY, radiative and non-radiative recombination lifetimes, initial carrier concentration for MNPs S1–S5. The radiation complex factor B: 10^{-10} cm^3/s.

MNPs	Lifetime (ns)	Relative PLQY	Radiative lifetime (ns)	Non-radiative lifetime (ns)	Initial carrier concentration (cm^{-3})
S1	5.37±0.02	0.78	6.8	24.4	1.5×10^{18}
S2	6.60±0.03	0.53	12.4	14.0	8.1×10^{17}
S3	9.96±0.07	0.47	21.2	18.8	4.7×10^{17}
S4	12.30±0.08	0.46	26.7	22.7	3.7×10^{17}
S5	14.21±0.50	0.20	71.1	17.7	1.4×10^{17}

The calculation of the carrier concentration

According to the Mott criterion defined as $n_{crit} = (\frac{k_B T}{E_B})/(11\pi r_B^3)$ [35] where, k_B is Boltzmann constant, T is the temperature, E_B is the exciton binding energy, r_B is the exciton Bohr radius. k_B=1.38×10^{-23} J/K, T=298.15K, E_B of MNPs S1–S5 are given in Table S1, r_B =2.8 nm.

The critical carrier concentration of MNPs S1-S5 are 6.4×10^{17}, 7.2×10^{17}, 9.2×10^{17}, 1.2×10^{18}, and 1.4×10^{18} cm^{-3}, respectively.
